# Contribution of Sequence Motif, Chromatin State, and DNA Structure Features to Predictive Models of Transcription Factor Binding in Yeast

**DOI:** 10.1371/journal.pcbi.1004418

**Published:** 2015-08-20

**Authors:** Zing Tsung-Yeh Tsai, Shin-Han Shiu, Huai-Kuang Tsai

**Affiliations:** 1 Institute of Information Science, Academia Sinica, Taipei, Taiwan; 2 Department of Plant Biology, Michigan State University, East Lansing, Michigan, United States of America; Ottawa University, CANADA

## Abstract

Transcription factor (TF) binding is determined by the presence of specific sequence motifs (SM) and chromatin accessibility, where the latter is influenced by both chromatin state (CS) and DNA structure (DS) properties. Although SM, CS, and DS have been used to predict TF binding sites, a predictive model that jointly considers CS and DS has not been developed to predict either TF-specific binding or general binding properties of TFs. Using budding yeast as model, we found that machine learning classifiers trained with either CS or DS features alone perform better in predicting TF-specific binding compared to SM-based classifiers. In addition, simultaneously considering CS and DS further improves the accuracy of the TF binding predictions, indicating the highly complementary nature of these two properties. The contributions of SM, CS, and DS features to binding site predictions differ greatly between TFs, allowing TF-specific predictions and potentially reflecting different TF binding mechanisms. In addition, a "TF-agnostic" predictive model based on three DNA “intrinsic properties” (*in silico* predicted nucleosome occupancy, major groove geometry, and dinucleotide free energy) that can be calculated from genomic sequences alone has performance that rivals the model incorporating experiment-derived data. This intrinsic property model allows prediction of binding regions not only across TFs, but also across DNA-binding domain families with distinct structural folds. Furthermore, these predicted binding regions can help identify TF binding sites that have a significant impact on target gene expression. Because the intrinsic property model allows prediction of binding regions across DNA-binding domain families, it is TF agnostic and likely describes general binding potential of TFs. Thus, our findings suggest that it is feasible to establish a TF agnostic model for identifying functional regulatory regions in potentially any sequenced genome.

## Author Summary

Identification of transcription factor binding sites based on sequence motifs is typically accompanied by a high false positive rate. Increasing evidence suggests that there are many other factors besides DNA sequence that may affect the binding and interaction of TFs with DNA. Through the integration of sequence motif, chromatin state, and DNA structure properties, we show that TF binding can be better predicted. Moreover, considering chromatin state and DNA structure properties simultaneously yields a significant improvement. While the binding of some TFs can be readily predicted using either chromatin state information or DNA structure, other TFs need both. Thus, our findings provide insights on how different histone modifications and DNA structure properties may influence the binding of a particular TF and thus how TFs regulate gene expression. These features are referred to as sequence “intrinsic properties” because they can be predicted from sequences alone. These intrinsic properties can be used to build a TF binding prediction model that has a similar performance to considering all features. Moreover, the intrinsic property model allows TFBS predictions not only across TFs, but also across DNA-binding domain families that are present in most eukaryotes, suggesting that the model likely can be used across species.

## Introduction

Transcription factors (TFs) regulate expression of target genes by interacting with specific TF binding sites (TFBSs). Because knowledge of TFBSs is crucial for understanding regulatory relationships between TFs and their target genes, a number of *in silico* TFBS identification approaches have been developed to complement expensive and time-consuming experiments [1]. However, TFBSs are usually short and highly degenerate, making them difficult to be identified [2]. In particular, TFBS identification based solely on the occurrence of specific sequence motifs is typically accompanied by a high false positive rate [3], and only a small subset of the large number of predicted sites are actually bound by TFs [4]. This high error rate in TFBS prediction is due to the fact that, in addition to sequence motifs, accessibility to the chromatin significantly influences whether a TF can interact with its binding sites [5,6]. For example, a large portion of predicted motif sites that are not bound by TFs tend to be located in chromatin inaccessible to nucleases [7,8]. Conversely, location of accessible chromatin tends to be correlated with experimentally defined TF binding regions [8–12]. Moreover, there is increasing evidence for association between chromatin accessibility and TF binding [13–16]. Chromatin state (CS), *i*.*e*. histone modifications and nucleosome occupancy [17,18], is a major determinant of chromatin accessibility and TF binding [19]. For example, histone acetylation weakens charge-dependent interactions between histones and DNA, thus increasing the chromatin accessibility for the transcription machinery [20]. Currently, chromatin accessibility mainly evaluated based on DNase I hypersensitivity has been used to distinguish genomic regions preferable for TF binding from regions that are not bound [9,21–23]. However, because chromatin accessibility is controlled by chromatin remodelers and histone modifications, it is expected that considerations in addition to DNase I hypersensitivity will be important to capture the influence of chromatin accessibility on TF binding. In addition to CS, TF binding affinity is affected by 3D molecular structure properties of naked DNA that directly interact with TFs [24] as well as genomic regions flanking the bound sites [25–27]. Some TFs recognize unique patterns of DNA structure independent of the specific sequences [28,29]. Therefore, most of the state-of-the-art TFBS identification methods supplement sequence motif models with either CS [7,30–35] or DNA structure (DS) [28,29,36–38] features. But no methods incorporate both CS and DS for TFBS prediction. Thus, the relative contribution of these features to binding of different TFs and how combining CS and DS may further improve TFBS predictions remains unclear. To investigate the impact of jointly considering CS and DS properties on the computational identification of TFBS, we systematically assessed how well 11 CS and 10 DS features were correlated with bound and unbound regions using a set of yeast chromatin immunoprecipitation (ChIP) data [39]. We next applied a machine-learning framework to evaluate the contributions of sequence motif, CS, and DS to TF binding prediction. Based on the prediction model, we further defined the most informative features required for identifying binding regions for each TF to determine how CS and DS may differentially influence TF binding.

Importantly, some aspects of chromatin states (e.g. nucleosome occupancy [40]) and DNA structure properties [41] can be predicted with genomic sequences alone. These features can be considered “intrinsic properties” of DNA, and in this study we show that the “intrinsic property model” has a comparable performance compared to the full model incorporating experimental data. It was recently proposed that, by integrating multiple datasets including the degree of conservation, transcript annotation, and histone modifications, a TF agnostic binding prediction model can be generated without using any TF-specific sequence motif information [42]. This raises the question whether a TF agnostic model can also be constructed by considering intrinsic properties. To evaluate the generality of the intrinsic property model, we applied the model to predict binding regions across TF domain families with divergent structural folds and binding mechanisms. Our findings indicate that it is possible to establish a TF agnostic model of regulatory region identification that works across TF families. Finally, to further demonstrate that the regions predicted to be TF bound by the intrinsic property model are biologically relevant, we tested whether target genes predicted to be bound by the same TFs tend to have more similar expression profiles, which would suggest co-regulation. Our finding is consistent with this expectation indicating that the predicted bound regions are relevant to transcriptional regulation and are relevant biologically.

## Results/Discussion

### TF-bound and unbound regions display distinct chromatin states and DNA structure properties

In addition to sequence motif (SM), chromatin state (CS) [7,30–33] and DNA structure (DS) [28,29,36–38] features have been shown to be informative for TFBS identification. However, earlier studies focused on either SM and CS features or SM and DS features. Thus the relative contributions of SM, CS, and DS features, both singly and in combination, towards TF binding prediction remains unclear. It is also not known if there are significant differences in how these factors influence prediction performance among different TFs. Knowledge of these differences has the potential to reveal varying molecular mechanisms of TF and TFBS interactions. To address these questions, we first examined the relationships between TF binding regions determined in genome-wide ChIP analysis of 40 TFs [39] and 23 features including two SM [43], 11 CS [44], and 10 DS [41] features (Table 1).

**Table 1:** The 23 features used in this study.

Feature [41,43,44]	Description
***Sequence motif (SM)*** ^***1***^	
*ScerTFhit*	The number of motif occurrences within an analyzed genomic region ^*2*^
*ScerTFpvalue*	The minimum *p*-value of sites identified by PWM scanning within an analyzed genomic region
***Chromatin state (CS)*** ^***3***^	
*H3*	Histone H3 occupancy
*ESA1*	Acetyltransferase ESA1 ChIP signal
*GCN5*	Acetyltransferase GCN5 ChIP signal
*H4ac*	Histone 4 lysine 5, 8, 12, and 16 acetylation
*H3K9ac*	Histone 3 lysine 9 acetylation
*H3K14ac*	Histone 3 lysine 14 acetylation
*H3K4me1*	Histone 3 lysine 4 methylation
*H3K4me2*	Histone 3 lysine 4 dimethylation
*H3K4me3*	Histone 3 lysine 4 trimethylation
*H3K36me3*	Histone 3 lysine 36 trimethylation
*H3K79me3*	Histone 3 lysine 79 trimethylation
***DNA structure property (DS) principle components*** ^***4***^	
*PC1 and ΔPC1*	DNA major groove geometry ^5^
*PC2 and ΔPC2*	Dinucleotide free energy
*PC3 and ΔPC3*	Dinucleotide twist and roll
*PC4 and ΔPC4*	DNA minor groove geometry
*PC5 and ΔPC5*	Dinucleotide tilt and rise

1.Values of the two sequence motif features were generated by scanning sequence with Position Weight Matrices from ScerTF [43].

2.p bound or unbound genomic region for a TF in question based on ChIP data

3.The 11 chromatin state features (CS) were obtained from Pokholok *et al*. [44]. The values for H3, ESA1, and GCN5 are averaged value over the analyzed genomic region.

4.The 10 DNA structure features (DS) were generated from principal component analysis (PCA, see Methods) on 125 DNA structure properties from DiProDB [41]. PC1-5: the average of principle component values over each target genomic region. ΔPC1-5: the difference between the average value of a particular principle component over a target genomic region and the average value of the same principle component over regions flanking the target.

5.The biological meaning was interpreted from top 10 dinucleotide properties having highest PCA loading coefficients.

**Fig 1:**
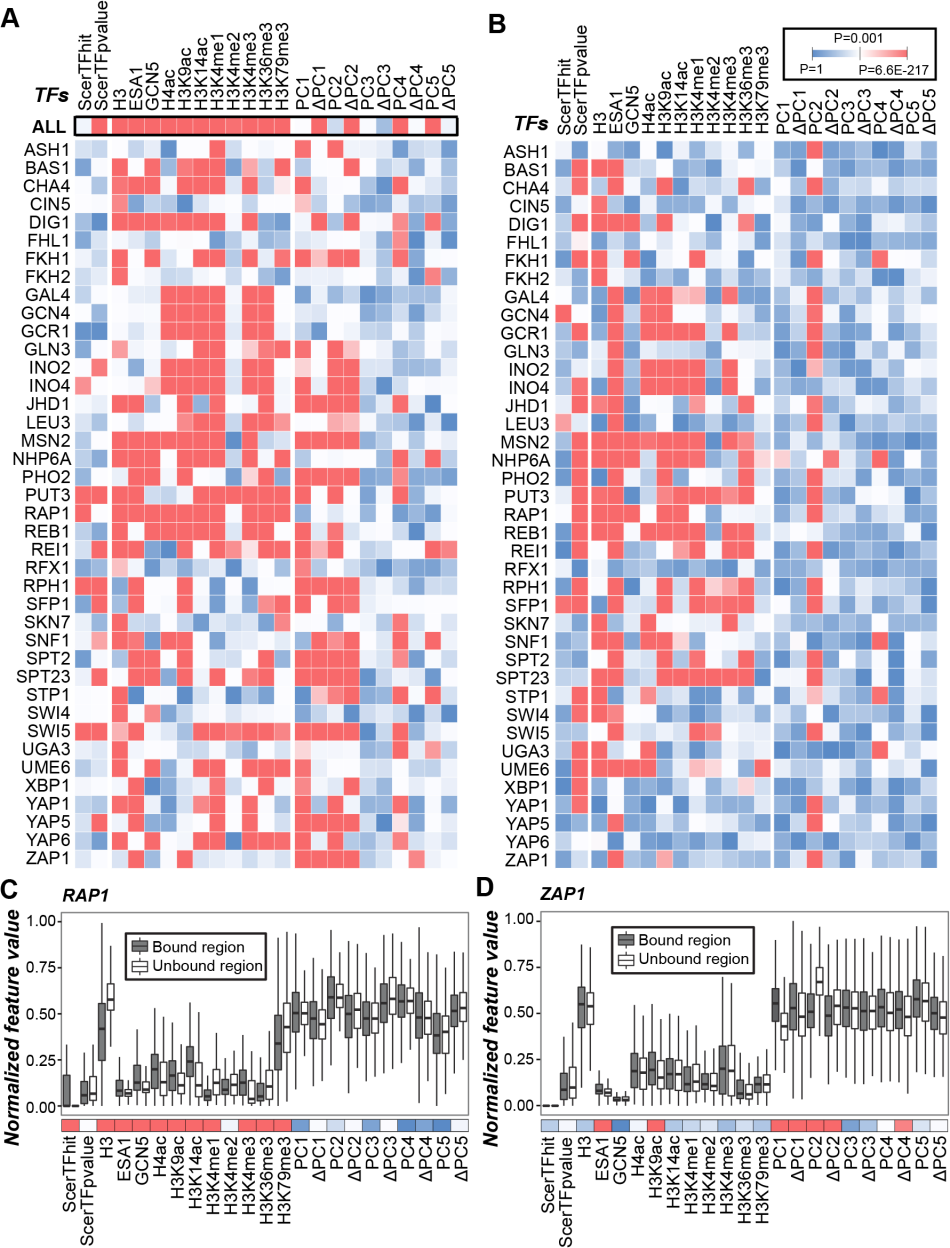
Evaluation of features distinguishing between bound and unbound regions and between regions bound by a single TF compared to the other TFs. *(A*,*B)* The *p*-values (color scale shown, adjusted by false discovery rate control for multiple testing) from two-sided Wilcoxon rank sum tests of differences in feature values (*A*) between bound and unbound regions of all the 40 analyzed TFs jointly (ALL) and separately, and *(B)* between bound regions of a single vs. the remaining TFs. The *p*-values for (*A*) and *(B)* are shown in S1 Fig and S2 Fig, respectively. *(C*,*D)* The value distributions of the 23 features for regions bound (black) and not bound (white) by (*C*) RAP1 and (*D*) ZAP1, respectively. The values were normalized into [0, 1] for each feature. The *p*-values of two-tailed Wilcoxon rank sum tests are shown below the boxplots: red, *p* < 10^−3^; white, *p* = 10^−3^; blue, *p* > 10^−3^.

When considering binding regions of all the TFs jointly, 16 of the 23 analyzed features showed significant differences between bound and unbound regions (two-sided Wilcoxon rank-sum test, *p*-values were adjusted by false discovery rate control for multiple testing, black rectangles in Fig 1*A*). Among SM features, the minimum *p*-values of motif-based predictions (*ScerTFpvalue*) are significantly smaller in bound compared to unbound regions (subsequently examined by one-sided Wilcoxon rank-sum test, *p* = 4.6×10^−11^). However, SM features have consistently higher *p*-values compared to all but one CS and three of the 10 DS features, indicating that CS and DS in general are better predictors of TF binding. For example, histone H3 (*H3*) occupancy, a proxy for nucleosome occupancy, was significantly lower in bound than unbound regions (subsequently examined by one-sided Wilcoxon rank-sum test, *p* = 1.2×10^−130^), consistent with the fact that bound regions tend to be exposed on the nucleosome surface [45] or tend to have lower nucleosome occupancy [46]. Our findings also agree with the notion that histone modifications can influence TF activities [47] as all histone mark related features have *p* values ranging from 4.6×10^−5^ (H3K4me2) to 5.6×10^−217^ (H3K4me1). Compared to CS features, the *p* values of test statistics of DS features tend to be higher, indicating DS features may play a less significant role in discerning TFBS. However, the differences in test statistics can be influenced by how CS and DS features were measured. Thus we cannot conclude whether CS is more important than DS or vice versa. Nonetheless, values of the DNA major and minor groove geometry related features (*ΔPC1* and *PC4*) and average dinucleotide free energy (*ΔPC2*) are significantly different between bound and unbound regions (two-sided Wilcoxon rank-sum test, highest *p* = 4.7×10^−8^; Fig 1*A*). Our findings are consistent with studies indicating that DS features such as the geometrical properties of the DNA major groove (*PC1*) and minor groove (*PC4*), and stability (*i*.*e*. free energy) (*PC2*) of the DNA helix could influence TF binding [48–50].

We next investigated the 23 features for each of the 40 TF individually (Fig 1*A* and *P*-values in S1 Fig). TFs differ greatly in which features are significantly different between bound and unbound regions. Taking RAP1 as an example, values for most of the CS features are significantly different between bound and unbound regions, yet none of the DS features is significant (Fig 1*C*). In contrast, for ZAP1, many DS but few CS-related features have significant test statistics (Fig 1*D*). However, some TFs, such as MSN2, REI1, SPT23, and SWI5, show significant differences both in CS and DS features (Fig 1*A*). Interestingly, values of five features (*ScerTFhit*, *PC1*, *PC2*, *ΔPC4*, and *ΔPC5*), which appear uninformative when considering binding regions of all TFs together, are significantly different between bound and unbound regions for a number of TFs. For example, the numbers of motif occurrences in regions of interest (*ScerTFhit*) are significantly higher in bound regions of INO4, PUT3, RAP1, RPH1, and SWI5 than unbound regions (all five TFs with *p*-values < 10^−3^, S1 Fig), indicating these TFs tend to bind to homotypic TFBS clusters [51,52]. In addition, the values of *ΔPC4* (DNA minor groove geometry) and *ΔPC5* (tilt and roll angles of dinucleotides) are significantly higher in bound than unbound regions for ZAP1 (one-sided Wilcoxon rank-sum test, *p* = 1.0×10^−4^) and REI1 (one-sided Wilcoxon rank-sum test, *p* = 9.1×10^−5^). These results suggest that, for these TFs, the differences in DNA minor groove geometry and tilt and roll angles of dinucleotides are likely important factors in binding site recognition.

In addition to comparing properties of bound and unbound regions, we were interested in determining how well may SM, CS, and DS features may allow us to distinguish bound regions of a TF vs. bound regions of other TFs. To this end, SM, CS, and DS feature value distributions between bound regions of a TF and bound regions of the remaining TFs were compared (Fig 1*B*, *P*-values in S2 Fig). We found that TFs differed the most in *ScerTFpvalue* indicating that differences in bound sequences is a dominant factor differentiating specific TF binding. Nonetheless, most CS features and one DS feature (*PC2*) are also significantly different between regions bound by different TFs. Taken together, our findings indicate that sequence specificity conferred by motifs, chromatin modifications, and/or DNA 3D structures may play distinct roles in determining binding specificity among these TFs, regardless of whether we compared bound vs. unbound regions or bound regions of one TF vs. the rest. In addition, CS and DS may be considered separately to identify binding regions of some TFs. But for others, these two feature sets should be simultaneously incorporated into a prediction model.

### Incorporating CS and DS features leads to improvement in TF binding predictions

**Fig 2:**
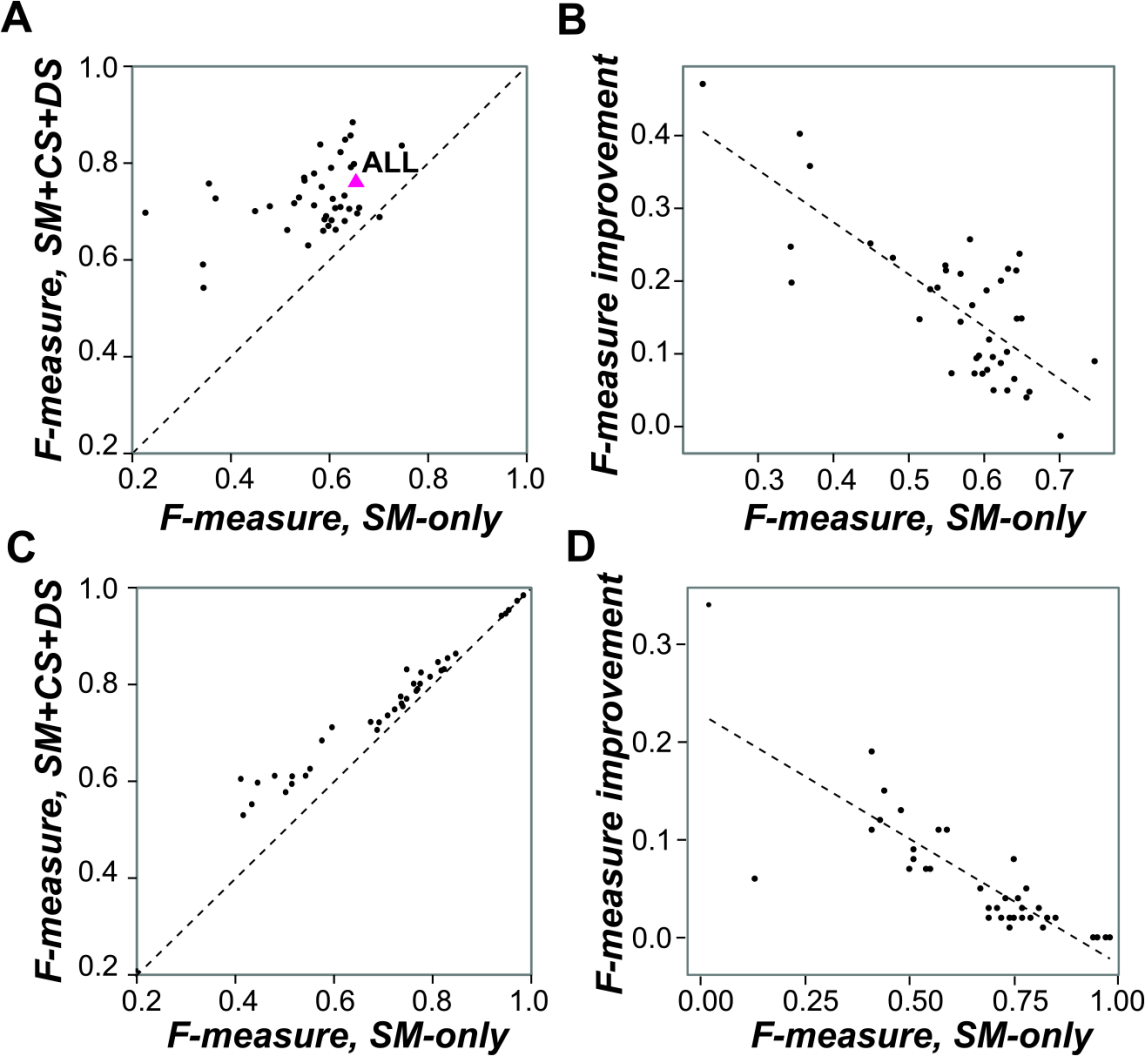
Performance improvement in binding region prediction models by incorporating chromatin state (CS) and DNA structure (DS) features. (*A*,*C*) The relationship between binding region prediction performance of models using sequence motif (SM) only and SM+CS+DS for each TF when contrasting *(A)* bound and unbound regions of a TF and *(C)* regions bound by one TF compared to regions bound by. the other TFs. The triangle indicates the average performance. The line indicates 1-to-1 relationship. (*B*,*D*) The relationship between the improvement in F-measure when incorporating CS and DS and the F-measures of random forest classifications using SM-only when contrasting *(B)* bound and unbound regions of a TF and *(D)* regions bound by one TF compared to regions bound by the other TFs.

Multiple SM, CS, and DS features are significantly distinct between bound and unbound regions of all TFs (Fig 1), indicating that they may be informative features for TFBS prediction. Earlier studies have shown that CS [7,30–33] and DS [28,29,36–38] can be incorporated for TFBS prediction, but they have not been considered together. To assess the utility of jointly considering CS and DS in TF binding prediction, we applied a machine-learning framework based on random forest [53] using these features to predict a genomic region would be bound by a specific TF or not. Random forest was chosen because: 1) it runs efficiently on large databases; 2) it systematically interprets the importance of each feature as well as their underlying relationships; 3) it is one of the most accurate learning algorithms currently available [53]. First we compared the performances of two random forest classification models: the first using only the two SM features (SM-only) and the second using all 23 features (SM+CS+DS) to determine the contribution of CS and DS features in binding region prediction. The performance of each classification model was measured using the averaged F-measure in ten independent runs, each with 10-fold cross-validation (Figs 2 and S3; other measurements including the area under Receiver Operating Characteristic curve (auROC), specificity, and accuracy are shown in S1 Table). F-measure is a value representing both precision (the proportion of predicted regions that are true binding regions) and recall (the proportion of true binding regions that are predicted). A perfect binding region classification model will have F-measure of 1, and a random model (with both classes, bound and unbound, equally likely) based on our dataset will have an F-measure of ~0.5 (for the relationship between F-measures and auROC values, see S4 Fig).

Among TFs, SM+CS+DS model F-measures (average = 0.73) are significantly higher than those of the SM-only model (average = 0.57; one-sided Kolmogorov–Smirnov test, *p* = 5.0×10^−14^), demonstrating that, overall, the prediction performance is significantly better when taking CS and DS into consideration (Fig 2*A*). In addition, considering CS and DS led to significantly better binding region predictions for every single TF (Figs 2 and S3). Nonetheless, there is a fairly large variance in the degree of improvement. For INO4 and SNF1, the F-measures increased 208% and 113%, respectively. On the other hand, for BAS1 and PHO2, the improvements were marginal (6% and 7%, respectively, S3 Fig). One possible reason for the marginal improvement is because the SM features alone may allow relatively good predictions. Consistent with this possibility, we found that the degree of performance improvement by incorporating CS and DS is significantly negatively correlated with the performances of the SM-only model (Pearson correlation coefficient (*r*) = −0.76, *p* = 9.5×10^−9^, Fig 2*B*). This finding further suggests that some TFs are likely CS and DS independent. However, in most cases TF binding is significantly influenced by CS and/or DS judging from the fact that incorporating CS/DS features improves binding region identification.

Similar conclusions can also be reached when predicting TF-specific binding by comparing bound regions of a single TF to bound regions of the other TFs (the one-TF-vs-rest model, Fig 2*C* and 2*D*). The average performance of the one-TF-vs-rest model when considering SM+CS+DS features is 0.73, the same as the performance of models based on bound/unbound regions (Fig 2). The F-measures for models considering CS/DS features are better than or equal to those of SM-only models because all values are either above or right on the diagonal line (Fig 2*C*). Similarly, there is a significantly negative correlation between the degrees of performance improvement when considering CS/DS features and the F-measures of the SM-only models (Fig 2*D*). These findings indicate that CS/DS features are particularly useful when models based on SM alone do not perform well for predicting binding regions. For these reasons, CS/DS features complement SM features in TF-specific binding prediction. Therefore, the contribution of CS/DS features in the one-TF-vs-rest model cannot be ignored. However, the performance improvement of the one-TF-vs-rest model by considering CS+DS features is not as pronounced compared to the improvement in the model differentiating bound/unbound regions. Thus to further reveal the contributions of CS/DS features to binding region prediction, in subsequent sections we focus on the models differentiating bound/unbound regions for each TF.

### The contribution of SM, CS, and DS to predicting binding regions is TF-specific

**Fig 3:**
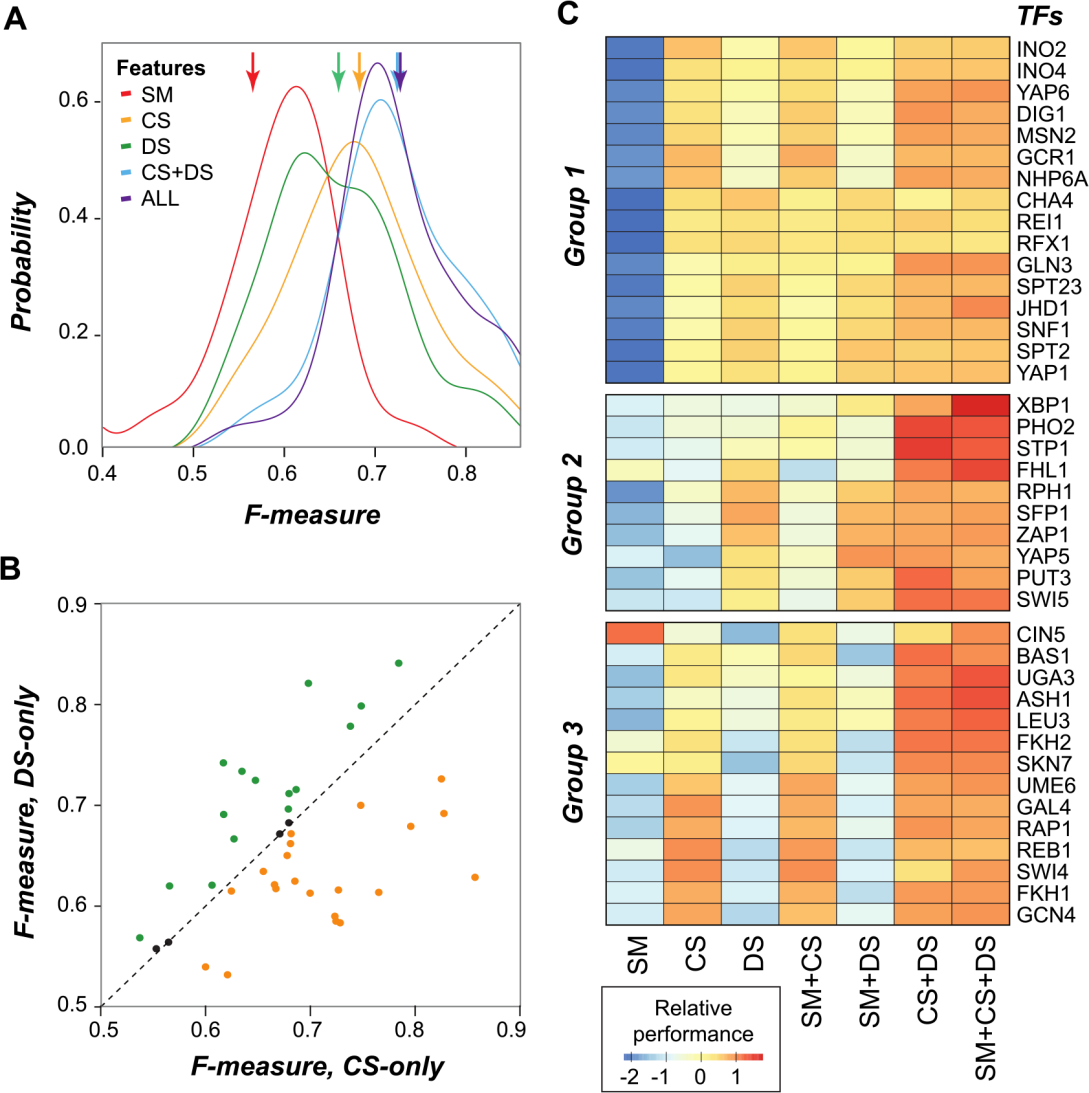
Contribution of SM, CS, and DS features to overall and individual TF binding region prediction. (*A*) The F-measure distributions of random forest classifications with different individual features or combinations of features. The y-axis indicates the probability with a specific F-measure. The arrowheads indicate the average F-measures. (*B*) The relationship between F-measures of binding predictions based on CS features only and DS features only. The dotted line shows the 1-to-1 relationship. Points in green (yellow) represent the TFs in which performance is better in the DS-only (CS-only) model. (*C*) Heat map showing the relative performance (*i*.*e*. standardized the F-measures to mean zero and variance one) in predicting binding region of each TF using individual features or combinations of features. The TFs are grouped into three classes: TFs with binding regions that can be predicted by either CS or DS (Group 1); TFs with binding regions that cannot be predicted well with only CS (Group 2) or only DS (Group 3) features.

To assess the contributions of SM, CS, and DS individually, as well as their combinations, to binding region predictions, seven random forest classification models were generated for each TF (Fig 3). We found that the prediction performance of the CS-only and DS-only models was significantly better than models using SM-only (Fig 3*A*, one-tailed KS test, *p* = 5.6×10^−7^ and *p* = 3.0×10^−4^, respectively). In addition, consistent with our finding that TFs differ greatly in their CS and DS value distributions (Fig 1), the performance correlation between the CS-only and DS-only models is rather weak (Fig 3*B*, *r* = 0.40, *p* = 1.1×10^−2^). This suggests that DS and CS features have significantly different contributions in the prediction of binding regions for different TFs. We also found that the performance of the CS+DS model is significantly better than CS-only or DS-only models (Fig 3*A*, one-tailed KS test, *p* = 7.5×10^−3^ and *p* = 7.3×10^−4^, respectively). Compared to the union of predictions by the CS-only and the DS-only models (32,274 sites correctly predicted by either the CS-only model, the DS-only model, or both), the CS+DS model correctly predicts only 528 more cases (S5 Fig). Thus it appears that, at least in the framework that we used, the effects of CS and DS are additive without a significant interaction term.

Based on the performance of the seven random forest classification models in predicting binding sites, the 40 TFs can be clustered into three groups (Fig 3*C*). For all groups, the best performing model considers both CS and DS feature sets. The main difference between these groups is which individual feature set is more important. Group 1 consists of TFs whose binding regions can be predicted by considering either CS or DS, whereas DS and CS are better predictors for Group 2 and Group 3 TFs, respectively. Although DS dominated in Group 2, the prediction performance was enhanced when CS features were included. However, CS features themselves contribute little to predicting binding regions of TFs in Group 2. In contrast, CS features dominated in Group 3, but jointly considering DS led to better predictions. However, in this case, DS features themselves were insufficient to predict Group 3 TF binding regions. In most cases, adding SM does not increase prediction performance.

Our findings suggest that Group 2 TFs may rely on variations within the DNA structure (indirect readout) rather than sequence base-specific recognition (direct readout) to recognize binding sites [54]. Furthermore, because CS features alone are not informative in predicting Group 2 TFs, their binding may not be regulated in a predominant fashion by chromatin state. Consistent with this notion, none of the TFs in Group 2 had reported interactions with histone modifiers [18]. In contrast, eight of the 14 TFs in Group 3 (ASH1, CIN5, FKH2, GCN4, REB1, SKN7, SWI4, and UME6) interact with histone modifiers [18], consistent with our findings that binding regions of Group 3 TFs are best predicted with CS features. Another line of evidence indicating the importance of histone modification in Group 3 TF binding region prediction is that six TFs in Group 3 (ASH1, CIN5, FKH1, FKH2, SKN7, SWI4) have strong histone modification signals around their TFBSs [30]. Experimental evidence also showed that one Group 3 TF, RAP1, competes with nucleosomes for DNA binding [17].

The observation of different TF subclasses echoes a recent study that classified TFs into pioneer, settler, and migrant classes based on DNase I footprints in bound regions [55]. Binding of settler TFs can be determined solely based on chromatin accessibility, whereas binding of migrant TFs is dependent on specific cofactor interactions. The pioneer TFs, on the other hand, can bind closed chromatin. Although the three TF groups (Fig 3) we defined differ in how chromatin state influences TF binding, it remains unclear how these groups may correspond to the TF classes Sherwood *et al*. [55] defined. This is because the groups we defined are based on relative contributions of various chromatin state features with no information on the mechanisms. But the Sherwood *et al*. [55] classes are defined based on mechanistic details of the interactions between TFs and chromatin. Taken together, our findings highlight the importance of simultaneously considering CS and DS in a TF binding region prediction model and that the contributions of CS and DS features in predicting binding regions are highly TF-specific.

### The binding region prediction model based on the three most important CS and DS features performs similar to the full model

**Fig 4:**
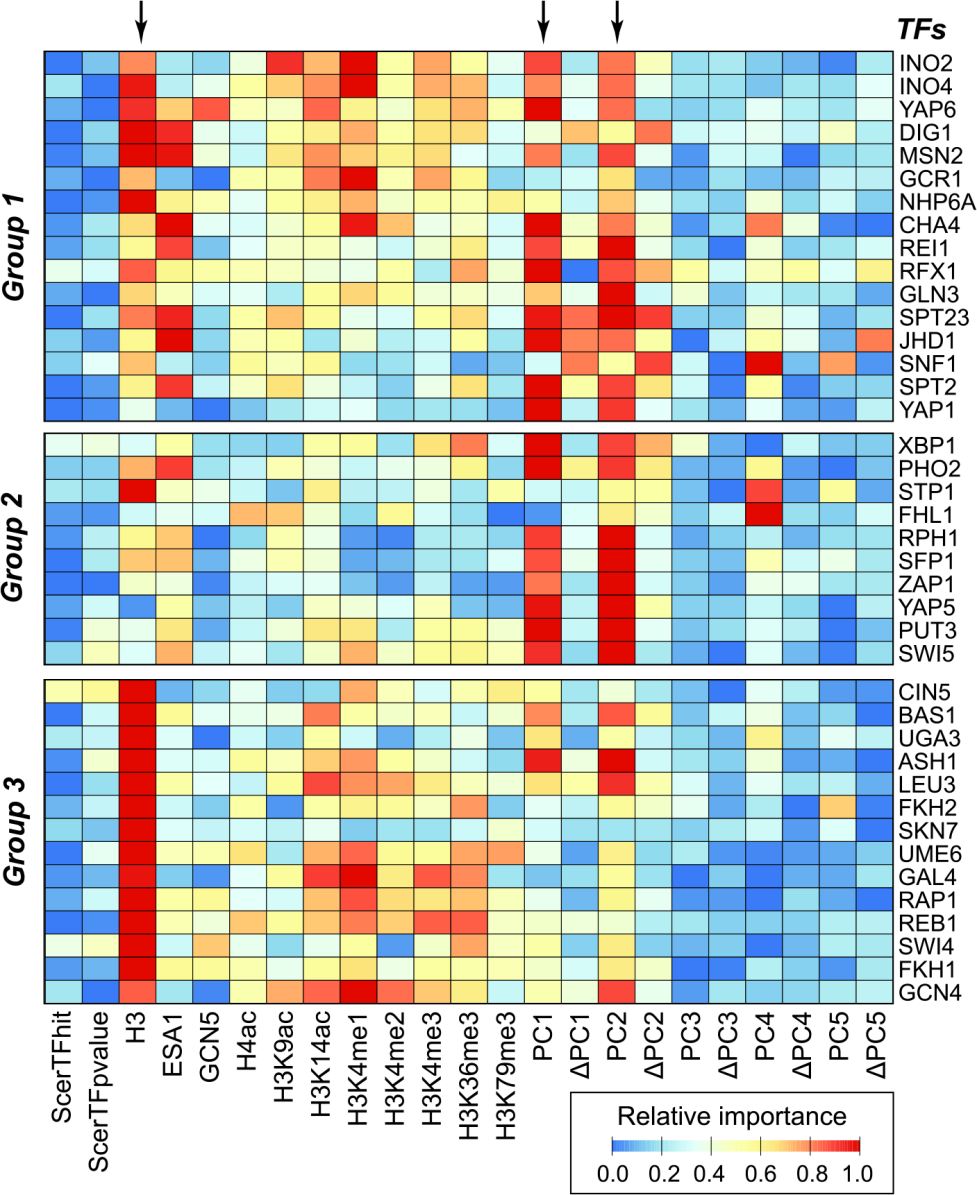
The relative importance of features for predicting binding regions of different TFs. Importance was defined as the decrease in accuracy after dropping a feature. The accuracy range was normalized to [0, 1] for each TF, where 0 is blue and 1 is red. The TFs were grouped into three classes as shown in Fig 3. Arrowheads indicate the most important features for predicting binding regions for most TFs.

Our findings indicate that DS and CS feature sets contain informative signals that can be used for TF binding region identification. Given that some of the 11 CS, 10 DS, and two SM features are better correlated with bound regions than others (Fig 1), a major question is what the relative contributions of each of the 23 features are to the binding region prediction models of each TF. To address this, an importance analysis was conducted to assess the relative importance of each feature. Importance is defined as the mean decrease in accuracy when the feature in question is removed. We found that the proxy for nucleosome occupancy (*H3*), DNA major groove geometry (*PC1*), and dinucleotide free energy (*PC2*) were the three most important properties for predicting binding for most TFs (arrows, Fig 4). The importance of nucleosome occupancy agrees with the observation that TF binding regions tend to be located in nucleosome depleted regions [45,46]. DNA major groove geometry (*PC1*) and dinucleotide free energy (*PC2*) are significantly correlated with DNA accessibility and stability, respectively, and both are known to affect TF binding [48–50]. Nonetheless, there is substantial variation in the relative importance of features among subsets of TFs. For example, H3K4me1 was particularly important for INO2, INO4, GCR1, CHA4, GAL4, and GCN4. In addition, the geometrical characteristics of DNA minor groove (*PC4*) were especially important for CHA4, SNF1, STP1, and FHL1.

Considering that *H3*, *PC1*, and *PC2* are particularly important for binding region predictions (Fig 4), we asked whether our binding region prediction model could be simplified by using these three major features alone. Remarkably, the performance of the simplified (3 features, average F-measure = 0.69) and the full (23 features, average F-measure = 0.71) models is not significantly different (two-sided KS test, *p* = 0.10; S6 Fig). In addition, the correlation between the performance of the two models is significant (*r* = 0.81, *p* = 1.7×10^−10^). Our findings indicate that nucleosome occupancy and the two DNA structure properties provide nearly sufficient information for identifying regulatory regions in a genome. It is important to note that the DNA structure properties used in the simplified model above are predicted from genomic sequences. On the other hand, the nucleosome occupancy data used above is experimentally derived. Given that nucleosome occupancy can be predicted with reasonable accuracy based solely on DNA sequence [40], next we asked if the *in silico* predicted occupancy can replace the experimental histone H3 occupancy data in TF binding prediction. The performance of the simplified 3-feature model with predicted nucleosome occupancy is comparable to the performance based on the same model but with experimentally derived nucleosome occupancy (S6 Fig, two-sided Wilcoxon rank-sum test, *p* = 0.35). We should note that the 3-feature model has reduced performance compared to the original 23-features model (S6 Fig, two-tailed KS test, *p* = 1.3×10^−2^). Thus some of the features excluded in the simplified model are clearly relevant. Nonetheless, considering that the simplified model use only features that can be predicted with genomic sequences alone, it performs surprisingly well. Given that the features used in the 3-feature model can be determined based solely on genomic sequences, they are referred to as “intrinsic properties”, and the simplified 3-feature model incorporating predicted nucleosome occupancy data is referred to as the “intrinsic property model”. Although the intrinsic property model does not perform as well as the full model, the intrinsic property model performs similarly as the experimental data-based 3-feature model and out-performs the SM-based model. The significance of our finding is that the three intrinsic property features can be calculated based on DNA sequences alone, highlighting the possibility of predicting binding regions simply using DNA sequences. In addition, the three intrinsic properties do not include SM-related features which traditionally have been used extensively for identifying binding sites using DNA sequence. It is intriguing that the model based on three purely computationally derived features significantly outperforms the traditional, SM-based model.

### The intrinsic property model allows cross-DNA binding domain prediction suggesting that a TF agnostic model can be established

To extend our finding beyond the 40 yeast TFs analyzed thus far, we used the intrinsic property model to predict binding regions for 161 yeast TFs that have ChIP data [39] but not sequence motifs in ScerTF [43]. We found that the intrinsic property model (average F-measure = 0.68) significantly outperformed a random predictor (average F-measure = 0.50, bootstrapping *p* < 1×10^−4^). The result indicates that our binding prediction model can be applied to TFs without relying on known binding sequence motifs. But we should note that the predictions we made here are based on the intrinsic property model that predicts general rather than specific TF binding. There are at least two explanations for our ability to construct a predictive model based on a subset of TFs. The first is that the training set contains TFs from multiple DNA binding domain families. Thus the model can be used to predict binding regions of the test set TFs in the same domain family. Alternatively, the intrinsic property model may be universal. That is, the model is general enough that knowledge of CS and DS feature measurements for some TFs is sufficient for predicting TFs with distinct structural folds and binding mechanisms.

**Fig 5:**
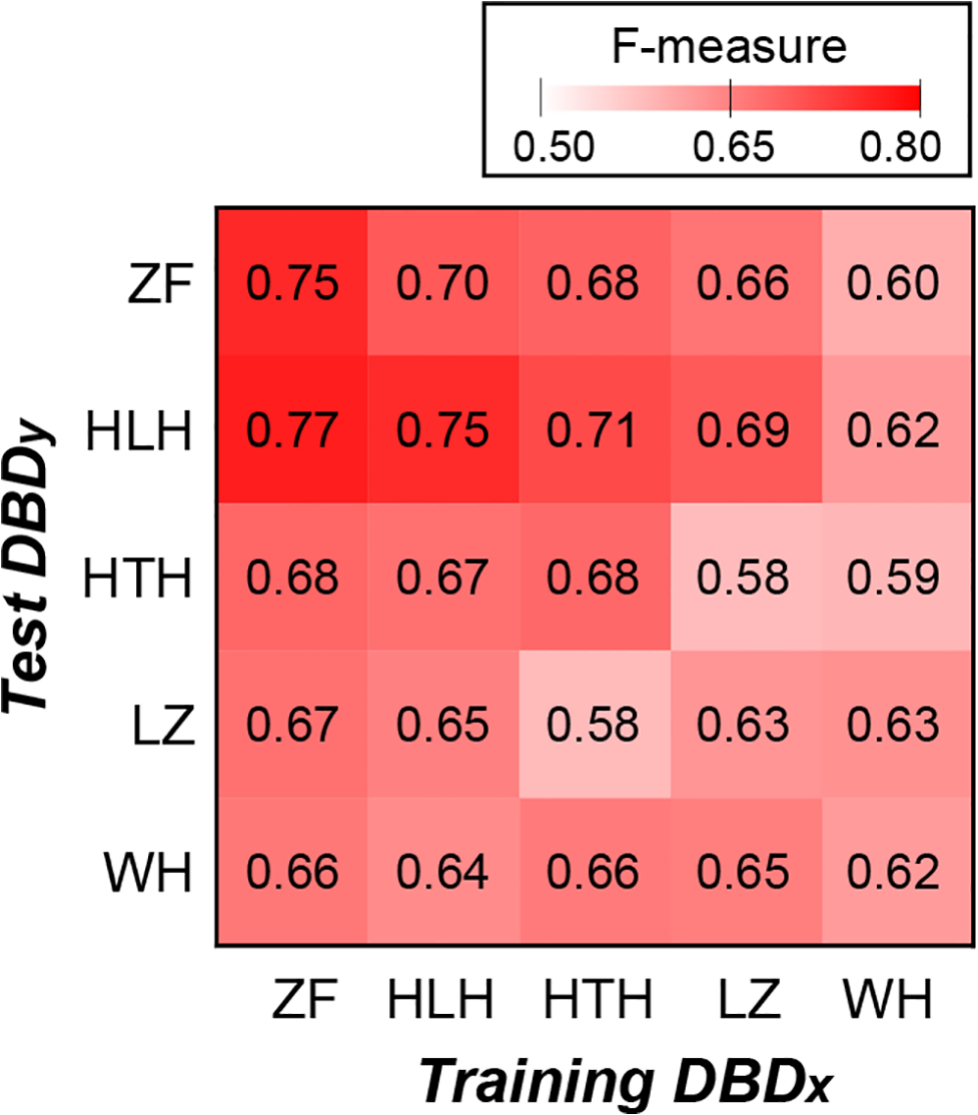
The performances of cross-DBD validations based on predictions using *in silico* predicted nucleosome occupancy, DNA major groove geometry, and dinucleotide free energy. The five DBD families examined were helix-turn-helix (HTH, 6130 sites), zinc finger (ZF, 8372 sites), leucine zipper (LZ, 3560 sites), winged helix (WH, 1070 sites), and helix-loop-helix (HLH, 2944 sites). Each value in the heat map is the F-measure of a model trained with the dataset of DBD_x_ family member binding regions to predict the test dataset consisting of binding regions of TFs with DBD_y_. The F-measures on the diagonal are obtained by 10-fold cross-validation.

To distinguish between these two possibilities, we conducted a cross-DNA-binding domain (DBD) study. First a model was trained with binding sites of TFs with a particular DBD. The DBD-specific model was then used to predict the binding regions of TFs containing another DBD. Five common types of DBDs were analyzed: helix-turn-helix, zinc finger, leucine zipper, winged helix and helix-loop-helix. The results showed that cross-DBD predictions have F-measures ranging from 0.58 to 0.77, which are all significantly better than random predictions (bootstrapping *p*-value < 0.0001) (Fig 5). Moreover, the performances of cross-DBD predictions are comparable to self-DBD predictions, and in three families cross-DBD predictions are actually better (Fig 5). Taken together, the intrinsic property model allows the prediction of TF binding regions in a non-DBD-specific manner, consistent with the interpretation that the intrinsic property model is sufficiently general to make reasonable predictions of TF binding regions for the additional 161 yeast TFs.

One question is that, if the contributions of SM, CS, and DS features to binding regions predictions differ greatly between TFs (Figs 3 and 4), how binding region prediction of a TF is feasible without using the binding data of the TF in question. There are two explanations. First, as shown in Fig 3C, TFs could be classified into three groups based on the contribution of SM, CS, and DS features in predicting TF binding regions. These groupings are feasible because some TFs apparently have similar SM/CS/DS feature values. This indicates that the binding regions of a TF, X, can be predicted based on consideration of other TFs with similar SM/CS/DS feature values (i.e. TFs in the same group as TF X). Second, one advantage of the random forest approach is in integrating multiple features and identify relevant combinations of decision cutoffs for various features. This approach is feasible even when each feature has only weak contribution to accurate prediction of binding regions of a TF. In our case, there are some similarities in SM/CS/DS features among subsets of TFs (which may or may not be in the same group, Fig 3*C*) and these similarities, although far from perfect, can be captured by the random forest method as indicated by the reasonable performance of our models (Fig 5).

Our result also suggests that a significant component of TF binding depends on simply these three intrinsic properties of genome sequence: *in silico* predicted nucleosome occupancy, DNA major groove geometry, and dinucleotide free energy. Thus, provided the genome sequence is available, it is feasible to establish a TF agnostic regulatory region identification model. Although the TF binding profile is known to be dictated in part by non TF-specific features [9,16,21], our intrinsic property model is the first computational model for describing general binding properties of TFs by integrating CS and DS features that are derived from solely genomic sequence. Notably, the intrinsic property model, trained by current data in the unicellular model budding yeast, was independent of cellular state. On one hand, this indicates that the intrinsic property model is sufficiently general and its prediction is not influenced by how the experiments were performed. On the other hand, it remains to be determined to what extent this intrinsic property model is applicable to predict binding in different cellular contexts.

The binding data we have analyzed so far are from ChIP-chip experiments that do not cover the entire yeast genome, although Venters *et al*. [39,56] indicated that the genome-wide regulatory maps derived from their ChIP-chip dataset (one of the dataset used in this study) are similar to those obtained from the high-density ChIP Affymetrix microarrays. To assess whether our findings may be influenced by the limitations in microarray resolution and genome coverage, we collected ChIP-seq data for five yeast TFs that overlap with our original 40 TFs with ChIP-chip data and where experiments were conducted under similar conditions [57–60]. For each TF, an intrinsic property model was trained by ChIP-chip data and then applied to distinguish between ChIP-seq peaks and random sequences. We found that the averaged performance is similar between models predicting ChIP-chip binding probes and those predicting ChIP-seq signal peaks (S2 Table and S8 Fig). Most importantly, results from one platform are not necessarily better than the other. Although there is still room for improvement, the ChIP-chip data are useful and our conclusions are not significantly affected by the type of data.

### Bound TFBSs identified by the intrinsic property model significantly lead to a coherent gene expression pattern

Identification of functional TFBSs that are bound by TFs and consequently impact gene expression has been one of the critical challenges in gene regulation studies [61]. Recently, several studies have addressed this challenge by integrating chromatin accessibility data [11,62,63]. However these associations between TFBS and chromatin accessibility have not yet been considered in predictive models of gene expression. In the predictive framework outlined in the previous section, we demonstrated the feasibility of using only intrinsic properties that can be calculated from genomic sequence to establish a model identifying functional TFBSs among sequence motif sites within the yeast genome. Next, we examined whether these TF binding regions predicted by the intrinsic property model are relevant in regulating the expression of target genes. The rationale is that genes containing regions predicted to be bound by the same TF would have a higher probability of being co-regulated by that TF and thus would display more highly correlated expression patterns under specific conditions compared to genes that are not predicted to be bound by the same TF. To determine if this is the case, time course expression data from four different “conditions” (cell cycle [64], galactose [65], mating [66], and nutrient deprivation [67]) were analyzed. Here the intrinsic property model provides a TF agnostic binding profile, which we used as a filter to distinguish functional TFBSs from the vast number of motif occurrences. We use only the intrinsic property model trained by the 40 TFs instead of the individual model for each TF; therefore, the binding data for each TF tested is unnecessary.

TFs regulating gene expression under each of these four conditions (referred to as condition-specific TFs) have been defined [68], and their Position Weight Matrices (PWMs) are available [43]. We first asked which genes contained motif sequences matching the PWM of each condition-specific TF in their promoter regions. Here the TFs analyzed do not include the initial 40 TFs used to establish the binding region prediction models. Note that the presence of a motif within a promoter is not definitive evidence of TF binding. Next, we classified each gene containing motif sequences of the TF in question into two gene sets. In the “bound” (or accessible) set, all positions of the motif sequences are within regions predicted to be bound by the TF based on the intrinsic property model (see Methods). In the “unbound” set, none of the motif sequence positions overlap with predicted bound regions. If the predicted bound regions are biologically relevant, we expect that genes in the bound set will have higher expression correlation under each condition. Consistent with this expectation, the gene expression patterns of bound sets tend to be more coherent than unbound sets (Table 2 and S7 Fig). These observations support our hypothesis that motif occurrences within predicted bound regions are more strongly correlated with gene expression than those within predicted unbound regions, demonstrating the feasibility of identifying authentic TFBSs using the intrinsic property model. Our results also show the advantage of using the intrinsic property model to identify functional TFBSs (*i*.*e*. it can remove unbound motif occurrences which are false positives).

**Table 2:** Expression patterns of genes with accessible motif occurrences are more highly correlated than those with inaccessible motifs.

Condition	TFs	#Time point ^1^	*P*-value ^2^
Cell cycle	GAT3, MBP1, MET4, MSN4, NDD1, STB1, STE12, SWI6, TEC1	18	3.58×10^−9^
Galactose	GAL4, MIG1, RGT1	26	1.58×10^−3^
Mating	MCM1, STE12, TEC1	7	1.06×10^−4^
Nutrient deprivation	DAL80, DAL81, DAL82, GAT1, GZF3, HAP2, MSN4, RTG1, RTG3	28	4.11×10^−9^

1. The number of time points in the time-series gene expression experiment.

2. The significance of difference between two correlation coefficient distributions (within-group pairwise correlations of bound and unbound sets) by one-sided KS test.

TF binding must have both a TF-specific as well as TF-agnostic components where the specific component dictates how different sites are bound by different TFs and the agnostic component is describing the general tendency of protein-DNA interactions. We found that, through integrating SM, CS, and DS features which have not previously been studied in combination, binding region prediction models can be established for both TF-specific and TF agnostic predictions. Our results demonstrate that models considering CS and DS features outperform a model considering SM features and highlight the importance of simultaneously considering CS and DS in yeast TF binding identification. Nonetheless, TFs differ greatly in which SM/CS/DS features allow prediction of their binding regions and can be classified into three distinct groups based on whether these features and/or combination of features are informative. The implication is that the specific primary sequence, chromatin accessibility, and 3D DNA structure contribute differently to the binding of these TFs to DNA. Thus, in addition to SM features, CS and DS features can also contribute to predictions of TF-specific binding. We also show that nucleosome occupancy, DNA major groove geometry, and dinucleotide free energy are particularly relevant features for TF binding prediction. Because these three features can either be predicted or calculated based on genome sequence alone, we refer to them as (DNA) intrinsic properties. Our analyses demonstrated the comparable performance of the intrinsic property model based on these three features to the model using all 23 SM, CS, and DS features. Most importantly, the intrinsic property model can be applied not only between TFs but across TF DBD families. Given that the DBD families analyzed are found in evolutionarily divergent eukaryotic lineages, our finding suggests that it is possible to construct a TF agnostic regulatory region prediction model that can potentially be applied to sequenced species. This is consistent with an early suggestion that a general TF binding model can be constructed by considering conservation, transcript annotation, and histone modifications [42]. Taking this one step further, our findings suggest that a reasonable TF binding region prediction is feasible based on properties that can be inferred directly from genomic sequences alone. Although there is room for further refinement, the intrinsic property model can serve to provide first pass prediction of potential TF-bound regions. These results not only highlight the importance of considering the features that can be extracted from genomic sequences in modeling binding in a TF agnostic fashion, but also highlight the contributions of both CS and DS features in predicting general TF binding. We expect that such a model can significantly contribute to a better understanding of transcriptional regulation, particularly in species with little or no regulatory genomic resources. This study focuses on the unicellular model budding yeast, and thus cell-type specific binding, which is an important question and challenge in multicellular eukaryotes, should be addressed in future studies.

## Methods

### Sequence motif, chromatin state and DNA structure features

The features used in this study are shown in Table 1. For each yeast TF, the PWM was downloaded from ScerTF [43]. We scanned the DNA sequence with PWMs using Matrix-scan, a program in the RSAT package [69], to identify putative binding sites (*p* < 0.001). Two sequence motif features were then extracted for each PWM: 1) the minimum *p*-value of sites matching the PWM and 2) the number of motif occurrences (*p* < 0.001).

Chromatin state features included histone H3 occupancy, binding data of two acetyltransferases (ESA1 and GCN5), and the signal levels of seven histone modifications (H4K5ac8ac12ac16ac, H3K9ac, H3K14ac, H3K4me1, H3K4me2, H3K4me3, H3K36me3, H3K79me3), with all experiments performed on YPD grown budding yeast [44]. The value of each feature used in our analysis was the average signal intensity of the ChIP-chip probes covering more than half of each analyzed region (60 bp bound or unbound genomic region for a TF according to TF binding ChIP data, see next section **Random forest classification**). All the chromatin state feature values were normalized to the level of histone H3 occupancy [44].

The DNA structure features include 125 conformational and thermodynamic dinucleotide properties collected from DiProDB [41] as of Aug. 2013. We first applied principal component analysis to reduce the data size and to consolidate overly similar DNA structure properties. The top five principal components (PC1 to PC5, S3 Table) that explain 83.3% of variance in DiProDB properties were used for subsequent analyses. The biological meaning of each PC was interpreted from top 10 DiProDB properties having highest PCA loading coefficients (i.e. the weight by which the variable should be multiplied to obtain the component score, indicating the correlation between a DiProDB property and a principal component). For example, PC1 is annotated as DNA major groove geometry because seven of the top 10 DiProDB properties are geometrical characteristics of DNA major groove (such as DNA major groove distance, DNA major groove width, and DNA major groove depth). We calculated two feature values for each DNA structure PC: (1) the average of the PC values of dinucleotides (step size of one base pair) for each analyzed genome region (*PC*_*x*_), and (2) the difference of the average PC values between each analyzed region and both its 5’ and 3’ flanking 30 bp regions (*ΔPC*_*x*_ *)*.

### Random forest classification: Training, validation and importance analysis

The prediction models of TF binding were generated by the random forest classification model [53] using the sequence motif, chromatin state, and DNA structure features. The training and testing binding data were from a genome-wide ChIP-chip study of 201 regulatory proteins in *S*. *cerevisiae* grown at 25°C in rich media [39]. For each TF, the bound probes on the tiling microarray (at a 5% FDR threshold) were used as the positive dataset and the same number of unbound probes (with the lowest signals) as the negative dataset. Because 75–300 bp DNA fragments were hybridized on the tiling microarray [39], only parts of the positive probe regions are actual binding sites. To further refine the regions used as the positive set, we searched from 240 bp upstream to 240 bp downstream of each probe region to obtain a narrower 60 bp region that contained the best hit of the sequence motif for the TF in question in its center. In addition, we filtered out any probe with missing values for any analyzed feature. TFs with less than 30 bound probes were not considered for random forest classification to avoid over fitting. Because we are interested in dissecting the relative contributions of SM, CS, and DS features, we could only focus on 40 TFs with annotated sequence motif information in both the ScerTF database [43] and in the ChIP-chip dataset [39]. An additional 161 TFs for which sequence motifs are not available (i.e. not in ScerTF or less than 30 bound probes in the ChIP-chip dataset) were used only for testing the performance of the developed model, and the testing data were simply defined as the bound probe regions at a 5% FDR threshold and the same number of unbound probes with the lowest signals.

We adopted a 10-fold cross-validation procedure repeated ten times to examine the performance and robustness of the random forest models. The two parameters of random forest, *n*_*tree*_ = 500 (the number of built trees) and *m*_*try*_ = *p* ^*1/2*^ (the number of randomly sampled features for each tree, in which *p* is the number of features), were used following the suggestion of Liaw *et al*. [70]. To evaluate the performance, we calculated F-measure, the harmonic mean of precision (True Positive / (True Positive+False Positive)) and recall (True Positive / (True Positive+False Negative)). In addition, auROC was calculated for comparison using the *ROCR* package in R [71]. We also measured the importance of each feature X as the reduction in accuracy after dropping feature X in the model. All the random forest analyses were conducted in R using the *randomForest* package [70].

To further predict TF-specific binding, the one-TF-vs-rest model was developed for each TF similar to the bound-unbound model except that the negative data was the regions bound by the other 39 TFs. For each TF, a one-TF-vs-rest model was evaluated by comparing the TF in question to each of the rest 39 TFs. For each TF, the 39 performance measures were then averaged and reported in Fig 2. Because the numbers of bound regions among TFs are similar, the above scheme allows us to avoid the extreme unequal sample size problem if we compared the bound regions of a TF (a small number) to the regions bound by any of the other 39 TFs (a large number). To evaluate the influence of the ChIP data platform type, we collected five yeast TFs with peaks identified in experiments conducted under similar conditions [57–60]. The positive dataset contains all 60 bp regions with ChIP peaks in its center. The negative dataset contains randomly chosen 60 bp regions without any ChIP peak.

### Assessment of the impact of bound regions predicted by the intrinsic property model on gene expression

We analyzed time-series mRNA expression data for four “conditions” including cell cycle [64], galactose [65], mating [66], and nutrient deprivation [67]. For each condition, we first identified binding sites of previously defined condition-specific TFs [68] (TFs which overlapped with the initial 40 TFs used to establish the binding region prediction models were excluded) by scanning PWMs from ScerTF database [43] with the tool Matrix-scan[69]. For each condition-specific TF, a gene containing its TFBSs (motif mapping *p* < 0.001) in its promoter region (defined as ≤1000 bp of intergenic region upstream of the transcriptional start site) was classified into one of the following three gene sets: 1) all TFBSs are within regions predicted by the intrinsic property model to be bound by the TF in question, 2) no TFBS is within predicted bound regions, and 3) some TFBSs are within predicted bound regions. We define the first two groups as bound and unbound gene sets respectively, and exclude the last group in the following analyses due to its ambiguity. For each condition, only TFs that have at least ten genes in both the bound and unbound sets are considered in this study.

To test the hypothesis that the within-group expression correlation of genes in the bound set is significantly higher than that of genes in the unbound set, we calculated the maximal single time-lagged (i.e. shifting the expression profile of one gene forward or backward one time point relative to the other gene) Spearman correlation [72] for each pair of genes within each set. Absolute values were taken to consider active and repressed effects simultaneously. Subsequently, we test if this distribution of correlation coefficients was significantly larger for bound set than unbound set by one-sided Kolmogorov-Smirnov test.

## Supporting Information

S1 Fig Features distinguishing bound and unbound regions. Shown are *p*-values (adjusted by false discovery rate control for multiple testing) from two-sided Wilcoxon rank sum tests of differences in feature values between bound and unbound regions of all the 40 analyzed TFs jointly (ALL) and separately. Red, *p* < 10^−3^; white, *p* = 10^−3^; blue, *p* > 10^−3^.
(EPS)

S2 Fig Feature distinguishing bound regions of one TF and the other TFs. Shown are *p*-values (adjusted by false discovery rate control for multiple testing) from two-sided Wilcoxon rank sum tests of differences in feature values between bound regions of a TF and other TFs. Red, *p* < 10^−3^; white, *p* = 10^−3^; blue, *p* > 10^−3^.
(EPS)

S3 Fig The performance (F-measures) of random forest classifications. Models using SM-only (grey) and all features (black) for each TF.
(EPS)

S4 Fig The relationship between F-measure and auROC values of the SM+CS+DS model for each TF. The dotted line indicates the linear regression line. The prediction performances measured in F-measure and auROC are significantly correlated in this study (Pearson correlation coefficient r = 0.98, linear regression [*auROC*] = 1.08 × [*F-measure*] with *r* ^2^ = 0.95).
(EPS)

S5 Fig Venn diagram illustrating the overlap among predictions of DS-only, CS-only, and DS+CS models. (*A*) Correct predictions. (*B*) Incorrect predictions.
(EPS)

S6 Fig The performances of three models with different feature combinations are comparable in predicting binding regions for 40 TFs. “All” indicates the model using all SM, CS, and DS features. “Major” indicates the 3-feature model using *PC1*, *PC2* and experimentally derived *H3*. “Intrinsic” indicates the intrinsic property model using *PC1*, *PC2*, and predicted nucleosome occupancy.
(EPS)

S7 Fig The pairwise correlation coefficients distributions of bound and unbound sets in four conditions: *(A)* Cell cycle, *(B)* galactose, *(C)* mating, and *(D)* nutrient deprivation. The y-axis indicates the probability density of observing a specific correlation coefficient. The vertical dotted lines indicate the average correlation coefficients.
(EPS)

S8 Fig The comparison of the maximum ChIP-seq intensity between the bound and unbound regions predicted by the ChIP-chip-based model. Shown are P-values from one-sided paired two-sample *t*-tests with null hypothesis to be rejected that the average intensity of bound regions is smaller than unbound regions. The significantly higher intensities for predicted bound compared to unbound regions (all *p* ≤ 7.2×10^−5^) supports the idea that our predictive models can identify bound regions revealed via ChIP-seq.
(EPS)

S1 Table The performance of random forest classifications using all the 23 features. (PDF)

S2 Table Performance of the intrinsic property models for predicting ChIP-seq peaks and ChIP-chip bound regions. (PDF)

S3 Table The top five principle components of DNA structure properties used in this study. (PDF)
