# Supplementary material for: Contribution of Sequence Motif, Chromatin State, and DNA Structure Features to Predictive Models of Transcription Factor Binding in Yeast
Source: PLoS Comput Biol. 2015 Aug 20;11(8):e1004418. doi: 10.1371/journal.pcbi.1004418 (PMC4546298; doi:10.1371/journal.pcbi.1004418)
Supplement: S1 Table — (PDF) [file pcbi.1004418.s009.pdf]

**S1 Table. The performance of random forest classifications using all 23 features**

| TF | F-measure | auROC[1] | Recall | Precision | Specificity[2] | Accuracy[3] |
|---|---|---|---|---|---|---|
| ALL | 0.76 | 0.83 | 0.76 | 0.77 | 0.76 | 0.76 |
| ASH1 | 0.68 | 0.75 | 0.67 | 0.70 | 0.69 | 0.68 |
| BAS1 | 0.70 | 0.76 | 0.68 | 0.72 | 0.70 | 0.69 |
| CHA4 | 0.68 | 0.75 | 0.69 | 0.67 | 0.68 | 0.69 |
| CIN5 | 0.69 | 0.75 | 0.70 | 0.68 | 0.69 | 0.69 |
| DIG1 | 0.78 | 0.87 | 0.78 | 0.78 | 0.78 | 0.78 |
| FHL1 | 0.66 | 0.68 | 0.65 | 0.67 | 0.66 | 0.66 |
| FKH1 | 0.71 | 0.78 | 0.70 | 0.71 | 0.71 | 0.71 |
| FKH2 | 0.79 | 0.88 | 0.77 | 0.81 | 0.80 | 0.79 |
| GAL4 | 0.75 | 0.81 | 0.77 | 0.73 | 0.74 | 0.76 |
| GCN4 | 0.73 | 0.79 | 0.75 | 0.72 | 0.73 | 0.74 |
| GCR1 | 0.73 | 0.80 | 0.75 | 0.71 | 0.72 | 0.73 |
| GLN3 | 0.77 | 0.84 | 0.76 | 0.78 | 0.77 | 0.77 |
| INO2 | 0.66 | 0.73 | 0.69 | 0.63 | 0.66 | 0.68 |
| INO4 | 0.70 | 0.77 | 0.73 | 0.67 | 0.69 | 0.71 |
| JHD1 | 0.88 | 0.94 | 0.91 | 0.86 | 0.87 | 0.89 |
| LEU3 | 0.73 | 0.79 | 0.72 | 0.73 | 0.73 | 0.73 |
| MSN2 | 0.71 | 0.76 | 0.69 | 0.74 | 0.72 | 0.70 |
| NHP6A | 0.84 | 0.92 | 0.82 | 0.86 | 0.85 | 0.83 |
| PHO2 | 0.71 | 0.77 | 0.69 | 0.72 | 0.71 | 0.70 |
| PUT3 | 0.68 | 0.72 | 0.66 | 0.71 | 0.68 | 0.67 |
| RAP1 | 0.80 | 0.87 | 0.82 | 0.78 | 0.79 | 0.80 |
| REB1 | 0.71 | 0.79 | 0.74 | 0.68 | 0.70 | 0.72 |
| REI1 | 0.70 | 0.77 | 0.68 | 0.73 | 0.70 | 0.69 |
| RFX1 | 0.54 | 0.59 | 0.56 | 0.53 | 0.55 | 0.55 |
| RPH1 | 0.73 | 0.80 | 0.73 | 0.73 | 0.73 | 0.73 |
| SFP1 | 0.82 | 0.89 | 0.81 | 0.84 | 0.83 | 0.82 |
| SKN7 | 0.66 | 0.69 | 0.64 | 0.68 | 0.66 | 0.65 |
| SNF1 | 0.76 | 0.83 | 0.75 | 0.76 | 0.76 | 0.76 |
| SPT2 | 0.72 | 0.79 | 0.70 | 0.74 | 0.72 | 0.71 |
| SPT23 | 0.79 | 0.85 | 0.76 | 0.83 | 0.81 | 0.78 |
| STP1 | 0.67 | 0.72 | 0.65 | 0.69 | 0.67 | 0.66 |
| SWI4 | 0.85 | 0.88 | 0.88 | 0.82 | 0.83 | 0.85 |
| SWI5 | 0.84 | 0.91 | 0.79 | 0.89 | 0.87 | 0.83 |
| UGA3 | 0.69 | 0.71 | 0.65 | 0.74 | 0.70 | 0.67 |
| UME6 | 0.86 | 0.93 | 0.87 | 0.85 | 0.85 | 0.86 |
| XBP1 | 0.63 | 0.66 | 0.64 | 0.62 | 0.63 | 0.63 |
| YAP1 | 0.59 | 0.60 | 0.57 | 0.61 | 0.58 | 0.57 |
| YAP5 | 0.71 | 0.75 | 0.67 | 0.75 | 0.71 | 0.69 |
| YAP6 | 0.71 | 0.76 | 0.72 | 0.70 | 0.71 | 0.72 |
| ZAP1 | 0.76 | 0.81 | 0.73 | 0.80 | 0.78 | 0.75 |

[1] Area under the curve of Receiver Operating Characteristic

[2] Specificity = True Negative / ( False Positive + True Negative )

[3] Accuracy = (True Positive + True Negative) / (True Positive + False Positive + True Negative + False Negative )
