# Supplementary material for: Contribution of Sequence Motif, Chromatin State, and DNA Structure Features to Predictive Models of Transcription Factor Binding in Yeast
Source: PLoS Comput Biol. 2015 Aug 20;11(8):e1004418. doi: 10.1371/journal.pcbi.1004418 (PMC4546298; doi:10.1371/journal.pcbi.1004418)
Supplement: S2 Table — (PDF) [file pcbi.1004418.s010.pdf]

S2 Table. Performance of the intrinsic property models for predicting ChIP-seq peaks and ChIP-chip bound regions.

TF	# Positive Cases		True Positive Rate		F-measure	
	ChIP-seq	ChIP-chip	ChIP-seq	ChIP-chip	ChIP-seq	ChIP-chip
**GAL4**	14	268	1.00	0.77	0.82	0.71
**GCN4**	502	396	0.61	0.61	0.62	0.62
**RAP1**	571	354	0.76	0.66	0.72	0.64
**REB1**	1776	930	0.61	0.6	0.62	0.61
**SWI4**	73	17	0.59	0.85	0.60	0.71
