# Supplementary material for: Contribution of Sequence Motif, Chromatin State, and DNA Structure Features to Predictive Models of Transcription Factor Binding in Yeast
Source: PLoS Comput Biol. 2015 Aug 20;11(8):e1004418. doi: 10.1371/journal.pcbi.1004418 (PMC4546298; doi:10.1371/journal.pcbi.1004418)
Supplement: S3 Table — (PDF) [file pcbi.1004418.s011.pdf]

S3 Table. The top five principle components of DNA structure properties used in this study

Dinucleotide	*PC1*	*PC2*	*PC3*	*PC4*	*PC5*
AA	-3.75	4.84	0.87	4.29	3.68
AC	-5.66	-1.89	0.91	-5.25	1.28
AG	-0.34	0.45	-7.64	-0.04	-1.02
AT	-7.81	2.78	-2.01	-3.45	-0.95
CA	8.01	2.22	1.67	-1.75	1.31
CC	1.15	-5.58	-2.82	3.45	-2.21
CG	8.35	-4.46	-1.66	-2.34	4.36
CT	-0.34	0.45	-7.64	-0.04	-1.02
GA	-0.68	0.54	3.48	3.75	-0.99
GC	-2.03	-8.27	8.02	-0.34	-1.67
GG	1.15	-5.58	-2.82	3.45	-2.21
GT	-5.63	-1.92	0.93	-5.38	1.25
TA	4.00	8.80	2.69	-2.65	-5.81
TC	-0.70	0.59	3.47	3.75	-1.00
TG	8.02	2.22	1.67	-1.75	1.31
TT	-3.75	4.84	0.87	4.29	3.68
Eigenvalue	5.0	4.5	4.0	3.4	2.6
Proportion of variance (%)	26	20.9	16.9	12.2	7.2
Cumulative variance (%)	26	46.9	63.9	76.1	83.3
Biological meaning [1]	Major groove geometry	Free energy	Twist and roll	Minor groove geometry	Tilt and roll

[1] The interpretation based on the top 10 DiProDB properties which have highest loadings in PCA (*i.e.* the weight by which the standardized original variable should be multiplied to get the component score, indicating the correlation between variable and principal component).
